# How to get started with a systematic review in epidemiology: an introductory guide for early career researchers

**DOI:** 10.1186/0778-7367-71-21

**Published:** 2013-08-07

**Authors:** Hayley J Denison, Richard M Dodds, Georgia Ntani, Rachel Cooper, Cyrus Cooper, Avan Aihie Sayer, Janis Baird

**Affiliations:** 1 MRC Lifecourse Epidemiology Unit, University of Southampton, Southampton, UK; 2 Academic Geriatric Medicine, University of Southampton, Southampton, UK; 3 MRC Unit for Lifelong Health and Ageing, University College London, London, UK

**Keywords:** Systematic review, Systematic review methods, Meta-analysis, Early career researchers, Evidence synthesis, Observational studies

## Abstract

**Background:**

Systematic review is a powerful research tool which aims to identify and synthesize all evidence relevant to a research question. The approach taken is much like that used in a scientific experiment, with high priority given to the transparency and reproducibility of the methods used and to handling all evidence in a consistent manner.

Early career researchers may find themselves in a position where they decide to undertake a systematic review, for example it may form part or all of a PhD thesis. Those with no prior experience of systematic review may need considerable support and direction getting started with such a project. Here we set out in simple terms how to get started with a systematic review.

**Discussion:**

Advice is given on matters such as developing a review protocol, searching using databases and other methods, data extraction, risk of bias assessment and data synthesis including meta-analysis. Signposts to further information and useful resources are also given.

**Conclusion:**

A well-conducted systematic review benefits the scientific field by providing a summary of existing evidence and highlighting unanswered questions. For the individual, undertaking a systematic review is also a great opportunity to improve skills in critical appraisal and in synthesising evidence.

## Background

Systematic review is a powerful research tool which aims to identify and synthesize all evidence relevant to a research question. It is an improvement on a standard literature review as it uses systematic methods to search for, assess and combine the evidence [1]. The approach taken is much like that used in a scientific experiment, with high priority given to the transparency (and reproducibility) of the methods used and to handling all evidence in a consistent manner.

Systematic reviews aim to include all literature that is relevant to the review question, no matter the direction or significance of the result, thus reducing bias and improving our confidence in the conclusions [2]. A well-conducted systematic review benefits the scientific community by providing a summary of existing evidence as well as identifying where knowledge is lacking [3]. In this way, systematic reviews are an important driver of research. Systematic reviews also provide decision-makers with synthesized, reliable information which can then be used in policy-making. For example, the Cochrane Collaboration [4] produces and maintains systematic reviews of the effectiveness of healthcare interventions which have been primarily assessed using randomised trials. Systematic reviews can also be used to combine the results of observational studies. This may help to highlight a future type of intervention to be included in a randomised trial [5], or to explore underlying aetiological questions looking at the association between risk factor(s) and the outcome of interest [6-8].

Systematic review is employed across a huge range of scientific disciplines, not only medicine, and may be used by researchers of all levels. Early career researchers (ECRs) may find themselves in a position where they decide to undertake a systematic review, for example a systematic review may form part or all of a PhD thesis. Systematic reviews are often a major piece of work, and may take considerable time to conduct. However, the returns from such a piece of work are potentially considerable because they summarize all of the evidence in relation to a particular question. There are other advantages, for example there is no collection of primary data which can be costly and time-consuming, and review work gives a potentially broad exposure to a certain topic as well as epidemiological research in general.

It is strongly advised that systematic reviews are carried out by at least two reviewers who work independently to screen abstracts, extract data and assess risk of bias, thereby reducing the chance of reviewer bias and increasing reliability. Those with no prior experience of systematic review may need considerable support and direction getting started with such a project. Therefore, we have aimed to put together a guidance article, aimed at ECRs, that sets out in simple terms how to get started with a systematic review. This is not a comprehensive guide, but rather a useful starting point encompassing signposts to other resources. The process of systematic review may be applied to many types of study, including both observational and trial designs, and where data is collected using quantitative or qualitative approaches. For the purposes of this guide, we will be focusing on the process of reviewing observational studies which have used quantitative methods. Reviewing qualitative research may involve quite different methodology to that presented here, so alternative resources for guidance on this topic are suggested. A good starting point is the Cochrane Qualitative and Implementation Methods Group website [9].

## Discussion

### Identifying the need for a systematic review

Before embarking on a systematic review, it is important to check that you will not be duplicating existing research. You will therefore need to perform a literature search specifically looking for a systematic review on your topic, as well as checking databases which prospectively record systematic reviews such as PROSPERO [10]. If you do find an existing review, you will need to consider if a further one is now warranted (for example, because relevant research has been published in the interim period) [11].

### Developing a protocol

Before you begin your systematic review, it is imperative that you first develop a protocol that is agreed by all reviewers. This is important for several reasons. Firstly, it focuses the purpose of the review and ensures all reviewers are agreed on this. It also establishes *a priori* how the review will be carried out, and ensures methodological consistency between reviewers. Finally, it serves as a useful reference during the review process, for example when screening articles it will be helpful to have details of the inclusion criteria to hand.

**Figure 1:**
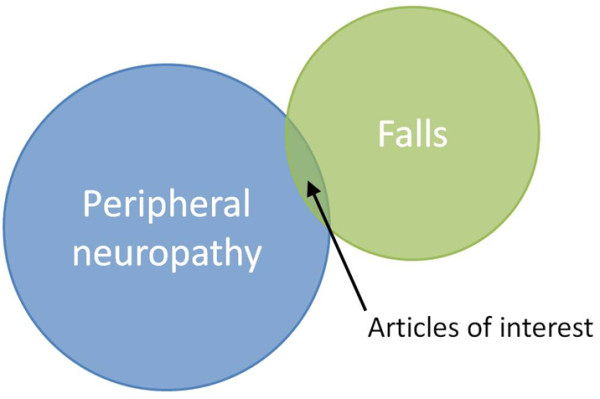
**Defining a search question:exposure/outcome model.**

Your protocol needs to contain enough information to enable all reviewers to be able to carry out the review in a consistent manner. The first fundamental step in designing a review protocol is to accurately define the main aims of the review together with some background information. The review question should be clearly stated, and may be one broad question or be broken down into smaller, more specific objectives. Take time to consider the review question when designing your search strategy. When considering aetiological questions, the search strategy will focus on information relating to the exposure and outcome which could be considered as the overlap between two different subjects. For example, searching for articles about the relationship between having peripheral neuropathy and then having falls as a consequence will produce a much smaller number of results than looking for either on their own (see Figure 1). This is referred to as an exposure/outcome model.

The methods section of the protocol will cover the whole process of the review. It should include details about the search strategy: which databases and years of publication are to be searched; what types of search terms will be used (see Table 1); how the screening will be carried out; and what other methods of obtaining data will be used (such as screening reference lists and contacting authors). Inclusion criteria for the review will relate directly to the review question, and should be outlined in sufficient detail that other researchers could apply them.

**Table 1:** Two main approaches to searching databases for articles


**MeSH (Medical Subject Headings) Terms**	
●	Denoted by a trailing slash, e.g. **Accidental Falls/**
●	The complete set of MeSH terms can be searched at http://www.nlm.nih.gov/mesh/
●	Usual to put the explode operator beforehand, telling the database that you want articles with the given term as well as terms in relevant sub-categories, e.g. **exp Accidental Falls/**
●	Those for the EMBASE database (“EMTREE”) include all of the MeSH terms
●	Look at terms assigned to relevant articles which you already have
**Free-text terms**	
●	Useful as there may not be MeSH term(s) relevant to the area of interest (also newer articles may not yet have been indexed with MeSH terms)
●	Denoted with double-quotation marks, a full stop and then the fields of interest, e.g. **“falls”.ti,ab** searches for this word in the title and abstract of all articles
●	Worth brainstorming synonyms (e.g. “peripheral neuropathy”, “peripheral sensory loss”) and also including alternative spellings (“haemoglobin”, ”hemoglobin” etc.)

Spend time over the inclusion criteria; they are a critical part of the review. Deciding on and laying out the criteria clearly before the review commences will ensure the correct papers are selected to answer your review question, and will save time later on. Factors you may want to consider are: the sample/population of interest; the independent variable; outcomes; study design and setting; language; and publication type. The methods section should also describe: how data extraction will be carried out; how the risk of bias of each article will be assessed; and how data synthesis will take place.

It is important to note that the protocol need not be set in stone. It is permissible to edit and adapt the protocol as you carry out the review, to make it more instructive and/or functional, provided all reviewers are in agreement and that you remain consistent in how articles are selected. It may be that you need to go back and change the inclusion criteria if the initial criteria resulted in inconclusive evidence to answer the review question. A log of protocol changes and reasons for the change should be kept in order that other researchers could repeat the review and arrive at the same answer. Lastly, it is worth referring to established systematic review guidelines at this stage, as it will help in designing your protocol and many journals require completed checklists on submission of the article. There are numerous useful guidelines, two examples commonly used are the PRISMA (Preferred Reporting Items for Systematic Reviews and Meta-analyses) [12] and MOOSE (Meta-analysis Of Observational Studies in Epidemiology) [13] guidelines.

### Searching

#### Searching databases

Your search strategy should involve searching relevant databases of published peer-reviewed literature. MEDLINE and EMBASE are the preferred primary sources, and both should be searched concurrently to ensure comprehensive coverage of the literature [14,15]. There may also be several other databases which are relevant to your review question; it is worth looking through a list such as that provided by the Centre for Reviews and Dissemination (CRD) to identify these. Having selected which databases to use, you then need to choose a search system to run your searches. It is often easiest to use the system which your institution’s library subscribes to, for example, OVID or ATHENS. You should also consider seeking the advice of an information specialist (again, perhaps via your institution’s library) when designing your search strategy.

**Figure 2:**
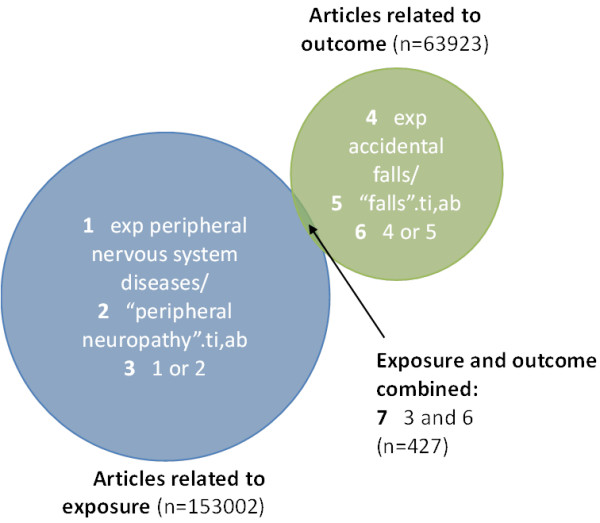
**Graphical representation of a search strategy to find articles relevant to peripheral neuropathy and falls.**

All articles in the MEDLINE database are assigned MeSH (Medical Subject Headings) terms by a team of coders. These provide a powerful way of finding articles related to a given topic. You can browse the different MeSH terms and find out more about the system on the US National Library of Medicine website [16]. It is important to also supplement your MeSH terms with free-text terms: relevant words or expressions found in specific parts of the article such as the title or abstract (see Table 1). The MeSH terms and free-text terms for each factor can then be combined using the “OR” operator. This produces large numbers of articles which can then be focused to the review question using the “AND” operator. See Figure 2 for a graphical representation of how this can be applied to a simple search looking for papers related to peripheral neuropathy (the exposure) and risk of having falls (the outcome).

Whilst it is important that your search is designed to capture everything of relevance, it should not be forgotten that the task of screening results needs to be manageable. For searches that have the potential to deliver a particularly large number of results, you may consider using additional criteria to limit the number of articles returned. For example, you could choose to only look at articles published after a certain date, or to exclude animal studies. This process is a balance: you need to weigh up the sensitivity requirements of the search versus the investment in terms of time-burden whilst screening. It is worth remembering that any restrictions you place on your searches will need to be considered when you assess the potential biases of your review whereby the less restrictions you place the better.

#### Grey literature

The term grey literature may be used to refer to any source of information which is not indexed in publication databases, for example conference abstracts or government reports. You could consider trying to identify relevant conference abstracts either by screening conference proceedings by hand or using a database such as the Zetoc database from the British Library [17]. The libraries of relevant centres may be able to help you to identify PhD dissertations. The Health Technology Assessments (HTA) database, available on the CRD website [18], is a useful source for identifying grey literature if your review question is related to healthcare. Searching for grey literature can be extremely time consuming, so this ought to be weighed up in terms of benefit to the review versus time burden.

### Handling and screening search results

#### Handling search results

The results of your searches will be in the form of a list of articles (and also conference abstracts or other types of grey literature if you have searched for these), which you will then need to screen for relevance to your review question. Depending on the topic and scope of your search, you may have a very large number of results to process, and you may well have more than one list if you have searched a number of relevant sources. In order to be able to screen these carefully and consistently, you will need an appropriate way of handling these data. It is best to store your results in an appropriate programme or format for screening. This could be a Microsoft Word document, a PDF file or reference management software such as Reference Manager or Endnote. A strength of using such reference management software is that it should be possible to remove duplicate articles where you have used several different sources for your search.

It will likely come down to personal preference as to how you manage your search results, but as with all processes in a systematic review, the key is to be consistent and to keep a record of your methods.

#### Screening search results

The purpose of screening is to identify sources of information that are relevant to your review question and fulfil your inclusion criteria. Therefore, in order to do this, it is important to refer back to your protocol to remind yourself of your inclusion/exclusion criteria. Screening should be conducted by two reviewers working independently and disagreements should be resolved through consensus with a third reviewer.

Screening of published articles has two main phases. The first is to look at the exported references from your search to identify potentially relevant papers. The second stage involves then getting a copy of these potentially relevant papers, reading them in full and making a decision on whether they meet the criteria to be included in the review.

Screening can be time consuming, but will generally get quicker as you become practiced at it. Make a note of how far you have got after every screening session, so you can be sure you are starting in the correct place when you return. Look at the title of the paper first; it is usually possible to make a quick decision as to whether the paper is relevant or not just from the title. If you need more information to make a decision, read the abstract. If you are still unsure, include it in the list of potentially relevant papers to obtain in full, and make a decision at that stage when you have the full paper in front of you.

The list of papers to obtain in full should contain enough information about each potentially relevant paper for you to identify it in a database again once you have finished screening. Record the title, first author, year of publication, and any number which identifies the source uniquely (such as the PubMed ID).

It is imperative that you keep a record of the numbers of papers included and excluded at each stage of the screening; this will be needed for when reporting your methods, usually presented in the form of a flow diagram. For the papers you get in full but then exclude from your review, you will need to record the specific reason for this as this will also be presented in the flow diagram.

Screening of conference abstracts is typically much quicker as there is no second phase of obtaining the full version as with a paper. However, as the information contained in conference abstracts is typically more limited, it may be more difficult to establish if they meet the inclusion criteria for your review which still need to be rigorously applied. A lack of information in a conference abstract might be one situation where you consider contacting the study author for more details, as described in the next section.

### Identification of additional sources following screening

#### Citation searching

There are two components to citation searching: 1. checking the bibliographies of papers already identified and included in the review, to screen earlier articles that they have cited, and 2. checking citation indexes (such as the ISI citation index through Web of Science), to screen later articles that have cited the included papers. Both methods are simple and quick, and can turn up previously overlooked, highly relevant literature.

#### Contacting authors

Contacting experts in the field is an important part of any search strategy. Following your initial database search, it may be worth considering contacting the authors of relevant studies (contact details of corresponding authors can usually be found on the published article). As these researchers have expertise in the relevant area of interest, they may be able to inform you of any articles or studies that have been missed (you will need to provide your current list of included articles). Remember, if any new articles are found by this route, the corresponding author(s) of these must also be contacted. While you are contacting authors, you may also wish to ask for clarity on some of the finer points of their article. However, consistency is essential, and all authors must be given the same opportunity to answer the same questions, or bias may be introduced into the review. Additionally, you may decide to ask authors to carry out some further analyses on the data presented in the article, to facilitate their inclusion in a meta-analysis. In addition to being as specific as possible in your request, it is important to make it simple to reply; perhaps provide a table for the authors to fill out. Be sure to acknowledge all help you receive from authors in the write-up of your review.

#### Unpublished data

You may also consider asking authors of your included studies, as well other experts in the field, if they are aware of unpublished studies that are relevant to your review. Similarly, you could list cohort studies which you think might have relevant data which have not previously been analysed or published, and then contact the cohort’s research team to see if they might be able to contribute to the review.

### Data extraction

Data extraction allows you to locate and synthesise the relevant information from the included papers. Using a standard data extraction form provides consistency and structure to this process. The form needs to be tailored to the individual review and the question that it is trying to answer. When designing the form, you need to think about what analyses you are hoping to do and what descriptive information and data you wish to present in the paper; this will help you to decide what information you wish to extract. It is also worth having a set of guidance notes that set out what is required in each section of the form. This clarifies any points that could be misinterpreted and ensures consistency in data extraction between reviewers.

The data extraction form should be piloted on a couple of studies that meet review inclusion criteria first. This will help you to assess its appropriateness for the review, and to ensure it facilitates effective extraction of the relevant data without including unnecessary information.

Be sure to include on your form: the paper title; authors; type of publication; year of publication; and any other citation information. It may also be useful to include where the paper was found, e.g. MEDLINE or other database search (along with any unique identifier or reference number for the article) or in the reference list of another paper and so on. The type of data extracted will depend on the review question and the types of study available. See Table 2 for a list of possible information that could be extracted.

**Table 2:** Information you may wish to consider including on the data extraction form (will vary depending on the research question being addressed)


**Study description**	
●	Aims and objectives of the study
●	Study setting (e.g. geographical location, time period)
●	Study design (e.g. cohort or case-control)
●	Recruitment procedures used
●	Inclusion and exclusion criteria
●	Length of follow-up
**Participant description**	
●	Baseline characteristics
●	Follow-up characteristics
●	Target population and final number of subjects studied for outcome
**Description of exposure (or intervention) and outcome measurements**	
●	Description of measurement of exposure and outcome (e.g. instrument, protocol, reliability)
●	Description of intervention, randomization and blinding (if applicable)
**Statistical data/results**	
●	Statistical techniques used (e.g. regression, t-tests)
●	Confounding factors adjusted for
●	Results of study analysis (e.g. direction and magnitude of association, precision)
●	Conclusions of study

### Risk of bias assessment

While extracting the data from your included papers, you should also carry out a risk of bias assessment (a process sometimes referred to as quality assessment). The purpose of this exercise is to assess the risk of bias in relation to your review question. This does not mean a critique of whether the authors themselves were biased. Instead, it refers to the possibility that the data are biased, based on flaws in the design or conduct of the study. This is an important process in the review because it will be useful during the synthesis process to be able to distinguish between studies which appear to give a reliable estimate in relation to your review question, and those studies where there appears to be a strong possibility that bias has been introduced.

Risk of bias assessment may take many different forms and there is no single, validated approach which is appropriate to all systematic reviews. You may wish to use a standard assessment tool, such as that provided in the Cochrane Handbook [19] or the RTI item bank [20], or you may prefer to develop one which is specific to the review.

### Results synthesis

#### Narrative synthesis

The initial task is to write a narrative synthesis of your results, often complemented by a series of tables. You will need to summarize information on the characteristics of included studies, such as study setting and design, exposure and outcome measure(s) used and what covariates were measured.

The next stage is to bring together the findings from your included studies. In the same way as for the results section of any individual scientific paper, the idea is to guide the reader through the key findings whilst trying to avoid interpretation. You might summarize the overall direction of effect of a group of studies and consider any results which are not consistent with an apparent trend. You could explore how study findings varied depending on your assessment of their risk of bias in relation to the review question. Following this, you can then explore the scope for meta-analysis.

#### Meta-analysis

Where you have results in numerical form, it is desirable to attempt to combine them using meta-analysis. Meta-analysis is a statistical technique for combining the findings of several independent studies, to provide a more comprehensive estimate of a measure of association. However, it may be that the included studies are too different to justify combining their results, for example in their outcome measure. Therefore, assessing whether meta-analysis is appropriate should be carefully considered [21].

Meta-analysis relies on having numerical estimates of the effect of the exposure(s) in question in a similar form, such as the change in your outcome per unit change in the exposure, or an odds ratio comparing the odds of the event of interest between exposed and unexposed groups. In general, it may be possible to carry out a meta-analysis using published results, or you may need to consider contacting authors to see if they can re-analyse their work in order to provide an estimate of effect that could be combined with those from other studies.

Such meta-analysis techniques are widely used for summarising evidence in systematic reviews but newer techniques are also being developed. For example, network meta-analysis is a statistical technique developed for estimating the relative effectiveness of different intervention arms in randomised controlled trials (RCTs) [22,23]. While variations on “conventional” meta-analysis may be valid and useful, they have their associated methodological challenges and it is always advised to consult a statistician on the appropriateness of any meta-analysis technique used.

There are several types of software that you can use to run your meta-analysis. General purpose statistical packages such as SPSS, SAS, R and Stata are widely used. For instance, commands such as the – metan – command in Stata provide highly flexible facilities for running meta-analyses and plotting out their results. However, with the exception of R (which is free) such statistical packages are often expensive to buy. A second option is to use software developed specifically for meta-analysis such as RevMan, Metawin and Comprehensive Metanalysis (CMA). These software packages may have a limited number of data formats that they can accept, or lack of flexibility in adjusting the graphical display of the results. A third option is to meta-analyse data using Microsoft Excel. Although it has a purchase cost, it is often installed in many computers as part of Microsoft Office. Using Excel is a very effective mechanism of learning meta-analysis since it allows you to familiarise yourself with the formulas. Neyeloff et al. 2012 have provided a detailed step-by-step guide about how to perform a meta-analysis and produce forest plots in a Microsoft Excel spreadsheet [24].

Whatever software you use, it is advisable that the data entry to create the dataset for meta-analysis is carried out by two reviewers independently to check agreement and avoid typing errors.

#### Sensitivity analysis and assessing bias

As well as generating a single pooled estimate, meta-analysis may allow you to explore whether an effect appears to be different in particular subgroups (sensitivity analysis). This can be done by categorising your studies in different ways and analysing their results separately. For example, you may decide in advance that you wish to explore whether an apparent overall effect differs by age group. This could be done by grouping studies by their mean age at time of assessment and producing a separate meta-analysis and pooled effect for each subgroup.

The output from a meta-analysis also includes the level of heterogeneity detected, which refers to the level of variation due to systematic differences in effect size between studies. The overall presence or absence of heterogeneity can be tested by the Q-statistic and can be quantified by the I^2^ value that shows the percentage of total variability attributable to between-study variation. If a high level of heterogeneity is detected, it is possible to group studies together based on their characteristics and see whether a particular aspect of study design or study setting seems to be contributing to the heterogeneity seen. The level of heterogeneity will also indicate the type of model to be fitted. If heterogeneity is absent, the most appropriate model is the fixed effects model that assumes an identical true effect across all studies. If heterogeneity is present, the appropriate model to fit is a random effects model that assumes that differences in effect size reported are due not only to sampling error but also due to systematic differences.

Even with a meta-analysis, you should still be critical in interpreting the result. A meta-analysis combines data, but if the original studies are biased, then clearly this bias will still be present in the meta-analysis result. Ignoring sources of bias may mean that the results of your review could be misleading. There are several ways you might attempt to assess the possibility of bias. For example, you could perform a sensitivity analysis in which you group studies into those which you have judged to have low and high risk of bias in relation to the review question, checking to see if there is a difference in the effect estimates. For more information on dealing with bias, see Turner RM et al. 2009 [25].

It is also recognised that studies showing a strong association or particular direction of results may be more likely to be both submitted and accepted for publication than those which do not, this is termed publication bias [26]. There are specific tests which can help to detect if negative study results might have been expected but are not included in your review because of publication bias [27]. Searching for unpublished data, as described earlier, has the potential to limit this source of bias in your findings.

### Useful resources

This paper is designed to be an introductory guide for Early Career Researchers, and is by no means a comprehensive manual for systematic review. We would advise you to seek guidance from colleagues with experience of systematic review and also to consult other guidance documents available. A useful publication is the Centre for Reviews and Dissemination (CRD) guidance for undertaking systematic reviews [2]. It presents in detail the methods and steps necessary to conduct a systematic review, as well as addresses questions relating to harm, costs, and how and why interventions work. The Institute of Medicine report Finding What Works in Health Care: Standards for Systematic Review recommends 21 standards for developing high-quality systematic reviews of comparative effectiveness research, and can be accessed from their website [21]. The Evidence for Policy and Practice Information and Co-ordinating Centre (EPPI-Centre), part of the Social Science Research Unit at the Institute of Education, University of London, has vast expertise in systematic review and can offer training and support. They also have some useful resources available to download for free from their website [28]. The National Institute for Health and Clinical Excellence (NICE) also has systematic review methodology documentation available on their website [29]. The Grading of Recommendations Assessment, Development and Evaluation (GRADE) Working Group is an international collaboration which has produced a substantial amount of guidance on assessing strength of evidence in health care, in the form of a toolbox and publications, both freely available from their website [30]. In addition to online sources of information, there are also numerous books available on the topic of systematic review (for example Petticrew and Roberts 2006 [3], Gough, Oliver and Thomas 2012 [31], Egger, Davey-Smith and Altman 2001 [32]). See Table 3 for some final, general tips.

**Table 3:** General tips


●	Do seek the advice of an experienced literature searcher, such as a member of staff in institution’s Library
●	Make sure you record each step in the review, much like keeping a log of laboratory experiments
●	Always think about consistency in your approach. Some parts of your review such as the protocol and search strategy may go through several iterations but the key is to handle all sources of information in the same way
●	Consider looking through existing published systematic reviews to get an idea of what the end result of your review may look like

## Conclusion

In conclusion, systematic review is a powerful and valuable tool in observational epidemiology, and can be used to answer research questions as well as generate new hypotheses and identify areas where knowledge is lacking. On a personal level, undertaking a systematic review is a great opportunity to improve skills in critical appraisal and in synthesising evidence, and can be useful through all stages of your career. We hope that this guide contains useful information and signposts for ECRs starting out on their first systematic review.

## Competing interests

The authors declare that they have no competing interests.

## Authors’ contributions

All authors read and approved the final manuscript.
